# Forsythiaside a facilitates autophagy to ameliorate chronic nonbacterial prostatitis in rats by blocking the PKCα/NF-κB pathway

**DOI:** 10.3389/fmolb.2025.1665650

**Published:** 2026-01-07

**Authors:** Xingwei Yu, Hongao Tan, Yunqiu Gao, Dandan Qiu, Yan Zhu, Haixin Qi

**Affiliations:** 1 Department of Urology, The First Affiliated Hospital of Zhejiang Chinese Medical University (Zhejiang Provincial Hospital of Chinese Medicine), Hangzhou, Zhejiang, China; 2 Department of Proctology, The First Affiliated Hospital of Zhejiang Chinese Medical University (Zhejiang Provincial Hospital of Chinese Medicine), Hangzhou, Zhejiang, China

**Keywords:** autophagy, chronic nonbacterial prostatitis, forsythiaside A, NF-κB pathway, protein kinase C alpha

## Abstract

**Background:**

Given the lack of effective treatment for chronic nonbacterial prostatitis (CNP) and the anti-inflammatory property of natural bioactive compound forsythiaside A (FTA), the therapeutic potential of FTA on CNP is worthy of investigation.

**Methods:**

CNP rat models were established using complete Freundʼs adjuvant, followed by a 4-week administration of FTA at different concentrations (40 and 80 mg/kg/d). The body and prostate of rats were weighed to calculate the prostatic index. Prostate damage and inflammatory infiltration were assessed using histological analysis and immunohistochemistry staining. Levels of inflammation-related cytokines, autophagic markers as well as the protein kinase C alpha (PKCα)/NF-κB pathway in prostate tissues were detected using enzyme-linked immunosorbent assay and western blot.

**Results:**

No significant change was observed in the body weight of CNP rat models administered with or without FTA. FTA treatment reduced the prostatic index and mitigated prostate damage and inflammatory infiltration of CNP rat models. FTA treatment decreased the number of CD3-positive cells and CD45-positive cells, while downregulating interleukin 1 beta (IL-1β), IL-2, IL-6, IL-17A, monocyte chemoattractant protein-1, and tumor necrosis factor alpha in prostate tissues of CNP rat models. FTA treatment promoted Beclin-1 and LC3B II/LC3B I expressions, and inhibited PKCα and p-p65/p65 expressions in prostate tissues of CNP rat models.

**Conclusion:**

FTA alleviates inflammation and facilitates autophagy in CNP rat models by blocking the PKCα/NF-κB pathway.

## 1 Introduction

Chronic nonbacterial prostatitis (CNP), belonging to category III prostatitis (chronic prostatitis/chronic pelvic pain syndrome), is a highly prevalent urological disease in men (Polackwich and Shoskes, 2016), constituting over 90% of prostatitis cases in clinical practice (Holt et al., 2016), with profound impacts on quality of life and mental health. Despite undefined etiology and pathogenesis of CNP, inflammation in response to autoimmunity has been confirmed to play a pivotal role in development of CNP and consequently affect prostate function (Motrich et al., 2018). Currently, pharmacotherapies (such as alpha-blockers and anti-inflammatory agents) remain the mainstay of clinical management for CNP (Datta, 2002; Chen et al., 2025). However, these strategies have limited and unsatisfactory therapeutic efficacy, underscoring the importance of developing new effective therapies.

Autophagy is a fundamental cellular process involving quality control, metabolism, and immunity (Parzych and Klionsky, 2014), playing a complex and context-dependent role in diseases. Autophagy dysregulation is implicated in various pathologies; for instance, excessive autophagy promotes apoptosis to aggravate non-small cell lung cancer, making it vital to precisely control autophagy (Wu et al., 2023). As a cellular degradative pathway, autophagy can protect cells from exogenous hazards and endogenous sources of inflammation (Deretic, 2021). Under normal physiological conditions, autophagy remains at a low level, and its dysfunction is often associated with dysregulated inflammation in human diseases (Deretic and Levine, 2018). In prostate pathology research, the focus has expanded from cancer to mechanisms such as cellular senescence (Li et al., 2023), yet the regulatory mechanism of autophagy in inflammatory conditions such as CNP remains unclear. Given the accumulating evidence underscoring the important role of autophagy in CNP, it is speculated that pharmacological modulation of autophagic activity could offer a compelling therapeutic candidate to halt the advancement of the condition (Zang et al., 2021). This discovery is particularly crucial, especially in the current era of research precisely targeting key signaling pathways (Wei et al., 2024).

Clinically, antibiotics, α-receptor blockers, non-steroidal anti-inflammatory drugs and phytotherapeutic agents are commonly used to treat CNP, but their effectiveness is variable (Tsunemori and Sugimoto, 2021). In recent years, traditional Chinese medicine has been reported to offer effective treatment for CNP with minimal side effects (Dong et al., 2021; Zang et al., 2021; Park et al., 2019). A representative example is Jiedu Huoxue decoction, a classical herbal formula composed of ten distinct Chinese medicinal herbs (Yan et al., 2020), exerting a strong anti-inflammatory effect on rats with CNP (Yan et al., 2019); however, its specific active component of the medicinal herb remains to be elucidated. Forsythiaside A (FTA) is a major bioactive component isolated from *Forsythia suspensa*, a medicinal herb that has long been used to treat various inflammatory conditions in China (Lee et al., 2018). FTA possesses a wide range of pharmacological activities including anti-inflammation, antivirus, anti-oxidative stress and neuroprotection (Gong et al., 2021). At present, although whether FTA exerts a therapeutic effect on CNP has rarely been reported, a growing number of studies have demonstrated that FTA can suppress the nuclear factor kappa B (NF-κB) pathway in several inflammatory diseases (Tong et al., 2021; Cheng et al., 2015; Pan et al., 2015). According to analysis of Swisstargetprodiction (http://swisstargetprediction.ch/), protein kinase C alpha (PKCα) is predicted to be the most probable target of FTA. PKCα is a member of the PKC family, which acts as cellular signal transducers and is involved in the modulation of inflammation (Leppänen et al., 2014). It has been reported that PKCα can activate multiple signaling pathways, including the NF-κB pathway, and plays a critical role in a variety of cellular physiological processes (Li and Luan, 2018).

This study was designed to explore the potential therapeutic effect of FTA on CNP using complete Freundʼs adjuvant (CFA)-induced animal models and to investigate whether FTA regulates autophagy to attenuate CNP via the PKCα/NF-κB pathway.

## 2 Methods

### 2.1 Animals and ethics statement

Six-week-old Sprague-Dawley rats (male, 240–260 g) were housed in a specific pathogen-free laboratory (12/12-h light-dark cycles) with free access to food and drinking water. All experimental procedures involving animals in this study were approved by the Institutional Animal Care and Use Committee of Zhejiang Baiyue Biology Technology Co., Ltd for Experimental Animals Welfare (NO. ZJBYLA-IACUC-20221202) and conducted based on the guidelines of the China Council on Animal Care and Use.

### 2.2 Drug preparation

FTA (C_29_H_36_O_15_, purity ≥99%, HY-N0028) and Gö 6983 (specific protein kinase C (PKC) inhibitor; purity: 99.32%, HY-13689) were purchased from MedChemExpress (Monmouth Junction, NJ, USA). Ten percent dimethyl sulfoxide (20-139, Sigma-Aldrich, St. Louis, MO, USA) was utilized to dissolve FTA and Gö 6983 to prepare stock solution. Chloroquine (CQ, autophagy pathway inhibitor; 60 mg/kg) was also procured from Sigma-Aldrich and diluted in sterile saline.

### 2.3 Animal experiments and administration

CNP rat models were constructed through an intraprostatic injection with CFA (YZ-642852, Acmec Biochemical Co., Ltd, Shnaghai, China), as described previously (Yang et al., 2019). In brief, rats were randomly assigned to eight groups: normal group, model group, model + FTA-L group, model + FTA-H group, model + CQ group, model + FTA-H + CQ group, model + Gö 6983 group, and model + Gö 6983 + FTA-H group. After being anesthetizing with 3% isoflurane (792632, Sigma-Aldrich, United States) in an anesthesia system (RWD Life Science, Shenzhen, China), rats from the last three groups were subjected to a lower abdominal incision to expose the prostate ventral lobes beneath the bladder. Subsequently, 100 μL CFA was injected into the exposed prostate, followed by wound suture. Meanwhile, rats from the normal group underwent sham surgeries without CFA injection. Next, rats from the model + FTA-L group and model + FTA-H group were administered with 40 mg/kg and 80 mg/kg corn oil-diluted FTA, respectively, through oral gavage on the second day after surgery (Lang et al., 2022). Concurrently, rats in the model + CQ and model + FTA-H + CQ groups received an intraperitoneal injection of CQ (Liu et al., 2024), while rats in the model + Gö 6983 and model + Gö 6983 + FTA-H groups received Gö 6983 (10 μg/kg) (Xu et al., 2018) via the same route. Gavage of high-dose FTA (80 mg/kg) in combination with the respective inhibitor was performed in the model + FTA-H + CQ and model + Gö 6983 + FTA-H groups. FTA administration was consecutively conducted for 4 weeks (Wang et al., 2017). The administration with the same volume of corn oil (vehicle) was conducted on rats from the model group in the same way. After that, all rats were weighed and then sacrificed under anesthesia (45 mg/kg pentobarbital sodium, P-010, Sigma-Aldrich, United States).

### 2.4 Calculation of the prostatic index

Prostate samples were excised from the sacrificed rats and weighed for calculating the prostatic index, as follows: prostatic index = prostate weight/body weight × 10000. After that, rat prostates were stored in liquid nitrogen until use.

### 2.5 Histological analysis

Rat prostates were fixed with 4% paraformaldehyde (PFD; abs9179, Absin, Shanghai, China), followed by dehydration. Then, the paraffin-embedded samples were sectioned into 5-µm thick sections. After deparaffinization and rehydration, the sections were stained with hematoxylin (abs9214, Absin, China) and eosin (AG1100, Acmec Biochemical Co., Ltd, China) at room temperature (RT) in sequence. A light microscope (Eclipse 80i, Nikon, Tokyo, Japan) was employed to observe prostatic morphology at × 40 magnification in five random fields. The degree of prostate injury was assessed using an inflammation score (0–4) scale, as per the guidance (Yang et al., 2019).

### 2.6 Immunohistochemistry (IHC) staining

To examine the presence of CD3^+^T cells and CD45^+^ leukocytes in rat prostate after CNP modeling, prostate sections were treated with citric acid (AC10801, Acmec Biochemical Co., Ltd, China) for antigen retrieval. Afterwards, the sections were exposed to 3% hydrogen peroxide (H299581, Aladdin, Shanghai, China), washed with phosphate-buffered saline (PBS; C0221A, Beyotime, Shanghai, China) and blocked with goat serum (abs933, Absin, China) at RT. Then, CD3 antibody (14-0030-82, Thermo Fisher, Waltham, MA, USA) and CD45 antibody (MA1-70000, Thermo Fisher, USA) were utilized to incubate the sections at 4 °C overnight. A hybridization with horseradish peroxidase (HRP)-conjugated secondary antibody (31430, Thermo Fisher, USA) was carried out at RT for 1 h. Immunoreactivity was detected by light microscopy (×40 magnification) after development with 3,3′-diaminobenzidine (AC11043, Acmec Biochemical Co., Ltd, China) and counterstaining with hematoxylin.

### 2.7 Enzyme-linked immunosorbent assay (ELISA)

To measure levels of inflammation-related cytokines including interleukin 1 beta (IL-1β), IL-2, IL-6, tumor necrosis factor alpha (TNF-α), IL-17A and monocyte chemoattractant protein-1 (MCP-1) in prostate of CNP rats, ELISA was performed using commercial rat ELISA kits (RLB00/R2000/R6000B/RTA00) from Novus Biologicals (Littleton, CO, USA) as well as commercial rat ELISA kits (D731078/D731095) from Sangon Biotech (Shanghai, China). Briefly, fresh prostate tissues were homogenized in pre-cooled PBS supplemented with protease inhibitor (G2008, Servicebio, Wuhan, China) and centrifuged at 5,000 × g for 10 min to obtain supernatant. Next, 100 μL diluted samples were cultured in each well of pre-coated ELISA plates at 37 °C for 90 min, and cultivated with 100 μL Biotinylated Detection Antibody for 1 h. After washing, the incubation with HRP Conjugate Diluent was carried out at 37 °C for 30 min, followed by a chromogenic treatment with Substrate Reagent without light. The absorbance in each well was measured at 450 nm with a microplate reader (Infinite 200, Tecan, Männedorf, Switzerland).

### 2.8 Western blot

Prostate homogenate was prepared with RIPA Lysis Buffer (89901, Thermo Fisher, USA) containing protease and phosphatase cocktail (P1045, Beyotime, China). Protein concentration in the collected supernatant was quantified using BCA Protein Assay Kit (71285-3, Sigma-Aldrich, United States). Protein extract was denatured and separated by 10% SDS-PAGE. Equal amounts of protein on the gel were transferred to polyvinylidene fluoride membranes (G6015, Servicebio, China) and blocked with 5% bovine serum albumin (A1933, Sigma-Aldrich, United States) at RT for 1 h. Next, membranes were incubated with primary antibodies against Beclin-1 (ab207612, 52 kDa, Abcam, Cambridge, UK), LC3B (ab192890, 16/14 kDa, Abcam, UK), protein kinase C alpha (PKCα; ab32376, 75 kDa, Abcam, UK), p-p65 (ab76302, 65 kDa, Abcam, UK), p65 (#8242, 65 kDa, Cell Signaling Technology, Danvers, MA, USA) and loading control GAPDH (ab181602, 36 kDa, Abcam, UK) at 4 °C overnight. The next day, the membranes were probed with HRP-conjugated mouse anti-rabbit IgG (D110065, Sangon Biotech, China) at RT for 2 h. The immunoblots were visualized with Enhanced Chemiluminescent Substrate (32106, Thermo Fisher, United States). Protein signals were densitometrically analyzed using Image Quant LAS 4000 system (GE Healthcare, Marlborough, MA, United States).

### 2.9 Statistical analysis

Data in this study are expressed as mean ± standard error of the mean from at least three independent experiments. Graphpad Prism 8.0 (GraphPad Software Inc., San Diego, CA, United States) was employed for all statistical analysis. Comparisons among multiple groups were analyzed using one-way analysis of variance. Difference with a *P*-value of <0.05 suggested a statistical significance.

## 3 Results

### 3.1 FTA treatment reduced the prostatic index of CNP rat models

**FIGURE 1:**
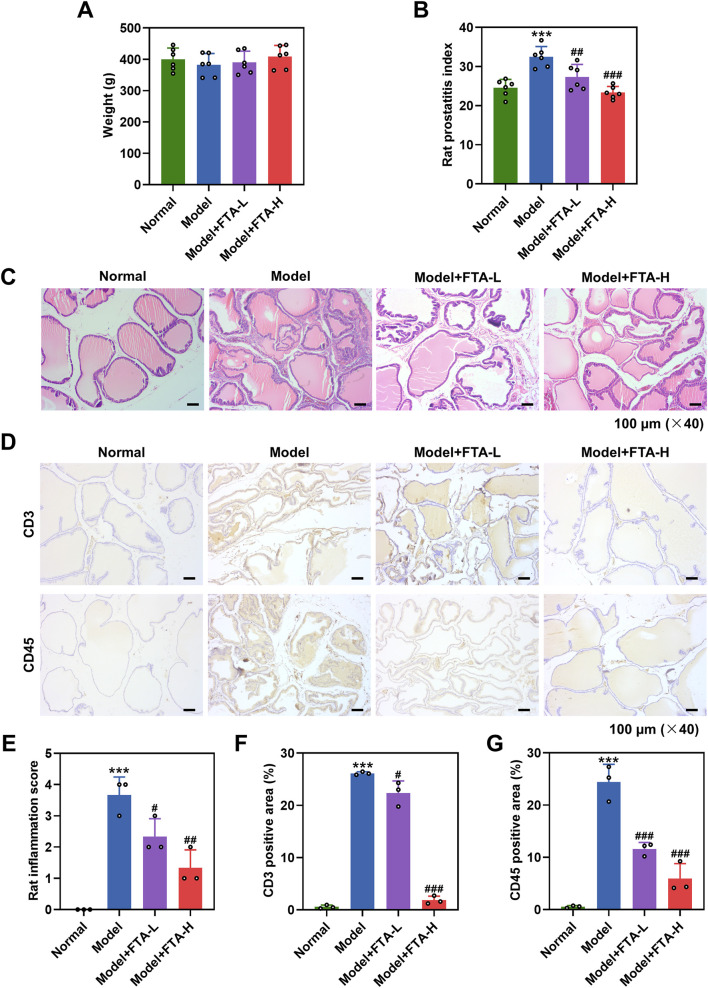
The attenuating effect of FTA on prostate damage and inflammation in CNP rat models. **(A)** CNP rat models were constructed by intraprostatic injection of complete Freundʼs adjuvant, followed by a 4-week oral administration with or without FTA (40 and 80 mg/kg/d). Weight changes in rats. **(B)** Prostatitis index changes in rats. **(C,E)** Representative images of prostatic morphology in rats after histological analysis (magnification: ×40, scale bar = 100 µm), and assessments of inflammation score. **(D,F,G)** Immunohistochemistry staining was used to detect CD3 and CD45 signals in prostate tissues of rats (magnification: ×40, scale bar = 100 µm). Data are shown as mean ± standard deviation. n = 6 for **(A,B)**; n = 3 for **(C–F)**. ^***^ *P* < 0.001, vs. normal; ^#^ *P* < 0.05, ^##^ *P* < 0.01, ^###^ *P* < 0.001, vs. model. Abbreviation: CNP, chronic nonbacterial prostatitis; FTA, forsythiaside A; L, low; H, high.

To investigate the effects of FTA on CNP, we first established CFA-induced CNP rat models. The body weight was barely changed between the normal group and the model group (Figure 1A). After 4 weeks of FTA administration, FTA at 40 mg/kg or 80 mg/kg had no significant effect on the body weight of CNP rat models (Figure 1A). The prostatic index of rats was elevated after CNP modeling (Figure 1B, *P* < 0.001), which was evidently diminished after 40 mg/kg or 80 mg/kg FTA treatment (Figure 1B, *P* < 0.01).

### 3.2 FTA treatment attenuated prostate damage and inflammatory infiltration in CNP rat models

In comparison with normal rats, significant inflammatory infiltration, edema and rough acinar shape were observed in the prostate of CNP rat models (Figure 1C), which were alleviated following 40 mg/kg or 80 mg/kg FTA treatment (Figure 1C). As shown in Figure 1E, the inflammation score was greatly increased in rats after CNP modeling (*P* < 0.001), but was decreased in the model + FTA-L and model + FTA-H groups (Figure 1E, *P* < 0.05). Next, we applied IHC assay to quantify expressions of CD3 and CD45 in prostate tissues to analyze lymphocyte infiltration. The results demonstrated that the number of CD3-positive cells and CD45-positive cells was increased in CNP rat models (Figures 1D, F, G, *P* < 0.001). Different concentrations (40 mg/kg and 80 mg/kg) of FTA treatment reduced the number of CD3-positive cells and CD45-positive cells in CNP rat models (Figures 1D, F, G, *P* < 0.05).

### 3.3 FTA treatment suppressed the release of inflammation-related cytokines in prostate tissues of CNP rat models

**FIGURE 2:**
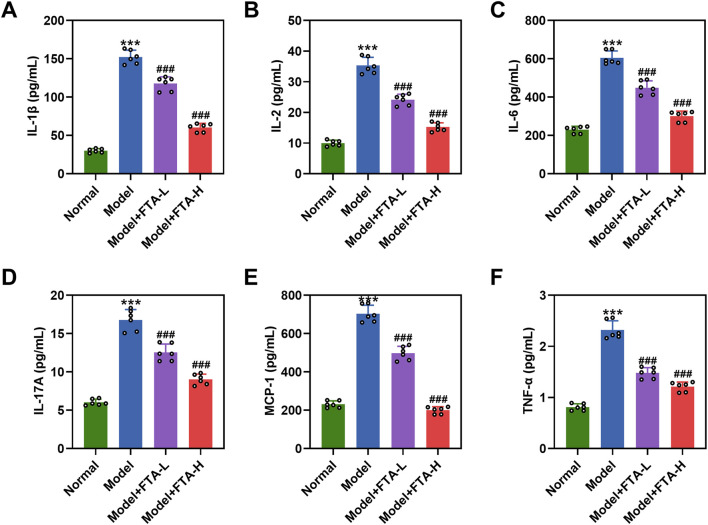
The suppressing effect of FTA on inflammatory-related cytokines in prostate tissues of CNP rat models. **(A–F)** CNP rat models were constructed by intraprostatic injection of complete Freundʼs adjuvant, followed by a 4-week oral administration with or without FTA (40 and 80 mg/kg/d). Enzyme-linked immunosorbent assay was performed to detect levels of IL-1β, IL-2, IL-6, IL-17A, MCP-1 and TNF-α in rat prostates. Data are shown as mean ± standard deviation. n = 6 in each group. ^***^ *P* < 0.001, vs. normal; ^###^ *P* < 0.001, vs. model. Abbreviation: IL-1β, interleukin 1 beta; IL-2, interleukin 2; IL-6, interleukin 6; IL-17A, interleukin 17A; MCP-1, monocyte chemoattractant protein-1; TNF-α, tumor necrosis factor alpha.

The release of inflammation-related cytokines in rat prostate tissues was assessed using ELISA. As demonstrated in Figures 2A–F, the expression levels of IL-1β, IL-2, IL-6, IL-17A, MCP-1, and TNF-α were significantly higher in the model group compared to the normal group (*P* < 0.001). Notably, treatment with FTA at doses of either 40 mg/kg or 80 mg/kg reduced the expression of these cytokines in the prostate tissues of CNP rat models. (Figures 2A-F, *P* < 0.001).

### 3.4 FTA treatment facilitated autophagy and blocked the PKCα/NF-κB pathway in prostate tissues of CNP rat models

**FIGURE 3:**
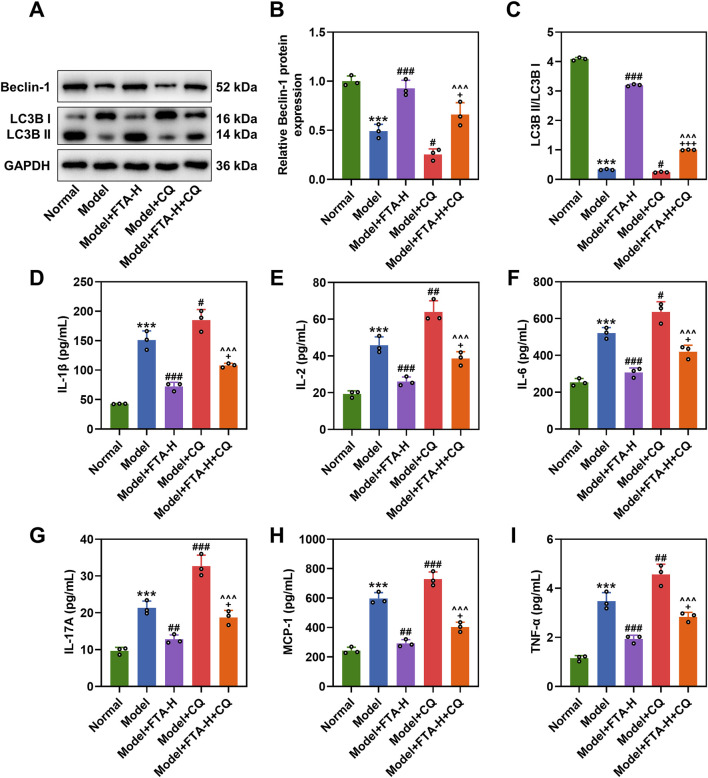
The regulatory role of FTA in autophagy in CNP rat models. **(A–C)** CNP rat models were constructed by intraprostatic injection of complete Freundʼs adjuvant, followed by a 4-week oral administration regimen with groups designated as normal, model, model + FTA-H, model + CQ, and model + FTA-H + CQ. Western blot was performed to measure protein expressions of Beclin-1, LC3B II/LC3B I, PKCα and p-p65/p-65 in rat prostates. GAPDH was used as a loading control. **(D–I)** Enzyme-linked immunosorbent assay was performed to detect levels of IL-1β, IL-2, IL-6, IL-17A, MCP-1 and TNF-α in rat prostates. Data are shown as mean ± standard deviation. n = 3 in each group. ^***^ *P* < 0.001, vs. normal; ^#^ *P* < 0.05, ^##^ *P* < 0.01, ^###^ *P* < 0.001, vs. model. ^+^ *P* < 0.05, ^+++^ *P* < 0.001, vs. model + FTA-H; ^^^*P* < 0.001 vs. model + CQ. Abbreviation: CQ, Chloroquine.

**FIGURE 4:**
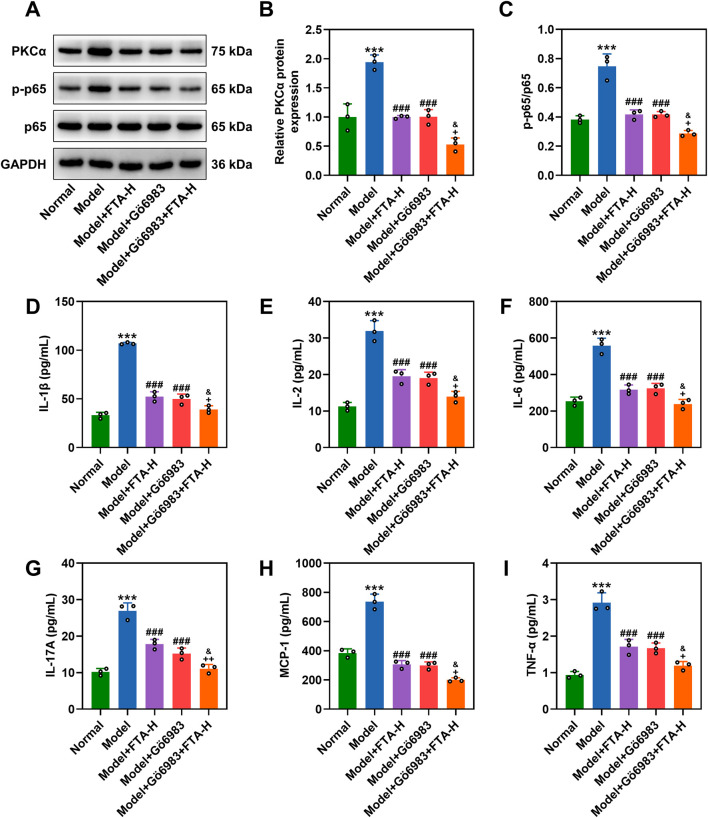
The regulatory role of FTA in the PKCα/NF-κB pathway in CNP rat models. **(A–F)** CNP rat models were constructed by intraprostatic injection of complete Freundʼs adjuvant, followed by a 4-week oral administration regimen with groups designated as normal, model, model + FTA-H, model + Gö 6983, and model + FTA-H + Gö 6983. Western blot was performed to measure protein expressions of PKCα and p-p65/p-65 in rat prostates. **(D–I)** Enzyme-linked immunosorbent assay was performed to detect levels of IL-1β, IL-2, IL-6, IL-17A, MCP-1 and TNF-α in rat prostates. GAPDH was used as a loading control. Data are shown as mean ± standard deviation. n = 3 in each group. ^***^ *P* < 0.001, vs. normal; ^###^ *P* < 0.001, vs. model. ^+^ *P* < 0.05, ^++^ *P* < 0.01, vs. model + FTA-H; ^&^ *P* < 0.05 vs. model + Gö 6983. Abbreviation: PKCα, protein kinase C alpha.

It is established that autophagy plays a critical role in regulating intercellular inflammatory response (Matsuzawa-Ishimoto et al., 2018). We subsequently detected autophagic markers including Beclin-1 and LC3B in rat prostate tissues using Western blot, and the results showed that CNP modeling downregulated Beclin-1 and LC3B II/LC3B I (Figures 3A-C, *P* < 0.001), but the expressions were increased due to FTA (80 mg/kg) treatment in prostate tissues of CNP rats (Figures 3A-C, *P* < 0.001). CQ inhibited the expressions of Beclin-1 and LC3B II/LC3B I, which was offset by FTA (Figures 3A-C, *P* < 0.05). ELISA results demonstrated that CQ promoted inflammation, but FTA attenuated its effect (Figures 3D-I, *P* < 0.05). Gö 6983 further blocked the PKCα/NF-κB signaling pathway (Figures 4A-C, *P* < 0.05). ELISA results showed that FTA reduced inflammatory factors in rats prostate tissues, while Gö 6983 further suppressed inflammation (Figures 4D-I, *P* < 0.05).

## 4 Discussion

In the present study, we firstly confirmed that FTA exhibited a therapeutic effect against CNP in rat by decreasing the prostatic index, attenuating prostate damage and reducing inflammatory response. Further, this study demonstrated that FTA promoted the activation of autophagy and inhibited the PKCα/NF-κB pathway in CNP rat-derived prostate tissues.

CNP is characterized by urogenital symptoms, such as urgency, frequent urination, pain and sexual dysfunction, in the absence of urinary tract infection or other identifiable pathogenic factors (Nickel et al., 2017). Existing evidence has verified inflammatory dysregulation in autoimmunity against prostate antigens as one of the contributors to CNP (Motrich et al., 2018). Therefore, animals injected with CFA intraprostatically, which induces inflammation and immune cell infiltration in the prostate gland due to T cell activation, are commonly used as experimental models of autoimmune prostatitis in CNP research (Kurita et al., 2018; Lin et al., 2017). In this study, rats injected with CFA exhibited significant prostate tissue damage, inflammatory response and increased numbers of CD3^+^T cells and CD45^+^ leukocytes, consistent with the findings of Yang et al. (Yang et al., 2019). However, these changes were reversed after FTA administration. As an active compound, FTA has been rarely studied in terms of its anti-inflammatory effect on chronic prostatitis. Intriguingly, recent evidence has shown that forsythoside B can significantly suppress inflammation to attenuate CFA-induced chronic inflammatory pain in mice by decreasing proinflammatory cytokines (IL-6 and TNF-α) (Wang et al., 2023). In ovalbumin-induced asthma, FTA has been found to reduce airway inflammation in mice (Qian et al., 2017). Additionally, FTA can decrease rat serum levels of proinflammatory cytokines (IL-6, IL-1β and TNF-α) to alleviate renal damage in adriamycin-induced nephropathy (Lu et al., 2020). In patients with CNP, immunoinflammatory cell-secreted proinflammatory cytokines, including IL-1β, IL-2, IL-6, MCP-1 and TNF-α, are upregulated in seminal plasma, prostatic secretions and urine (Breser et al., 2017). To our knowledge, MCP-1 acts as a critical mediator during the development of CNP through recruitment of T cells, monocytes and macrophages (Quick et al., 2012). IL-17A, produced by Th17 cells, is increased in CNP rat models, and the upregulation of IL-17A is associated with autoimmunity and inflammatory response (Peng et al., 2021). According to the results of ELISA, we found increased levels of IL-1β, IL-2, IL-6, MCP-1, TNF-α and IL-17A in prostate tissues of CNP rat models, but these tendencies were reversed by FTA treatment. Collectively, FTA could be used as a potential anti-inflammatory agent for treating CNP.

Subsequently, this study further investigated the underlying molecular mechanism of FTA on CNP. Intriguingly, the anti-inflammatory activity of FTA in many inflammatory disorders is associated with the regulation of the NF-κB pathway (Tong et al., 2021; Wang et al., 2022). NF-κB is a critical transcription factor that can be activated through various pathways to mediate transcription of key genes involved in inflammation, apoptosis or autophagy (Lee et al., 2018; Park and Koh, 2019). Activation of NF-κB in prostatitis has been demonstrated to be associated with chronic inflammation and disease severity (Paulis, 2018). Recently, it has been reported that inhibition of NF-κB by Qianliexin capsule can ameliorate 17 β-oestradiol-induced CNP by suppressing NLRP3 inflammasome (Zang et al., 2021). As a cytosolic multi-protein complex, NLRP3 inflammasome plays a regulatory role in autophagic process to balance host defense inflammation and prevent excessive inflammation (Biasizzo and Kopitar-Jerala, 2020). Lang et al. found that FTA can suppress the activation of NLRP3 inflammasome to attenuate methotrexate-induced intestinal mucositis in rats (Lang et al., 2022). In this study, the results of western blot revealed that expressions of Beclin-1 and LC3B II/LC3B I were decreased in prostate tissues of CNP rat models, confirming suppressed autophagy in CNP (Lu et al., 2019). It is well accepted that p65 activation is a central event in the NF-κB signaling pathway (Cui et al., 2014). In prostate tissues, we found that CNP modeling-induced downregulation of Beclin-1 and LC3B II/LC3B I as well as upregulation of p65 phosphorylation were reversed after FTA treatment. As a putative target of FTA, PKCα has been reported to play a regulatory role in inflammation (Guo et al., 2017), but its effect on chronic prostatitis is unknown. Chen et al. have suggested that PKCα inhibition protects the lung of mice against sepsis-induced hyperinflammatory response and oxidative stress, which could be accomplished by blocking the NF-κB pathway (Chen et al., 2022). PKCα also can stimulate the activation of NF-κB pathway through p65 nuclear translocation to regulate apoptotic resistance and thereby contribute to urothelial cell carcinoma (Zheng et al., 2017). In this study, we observed that the expression of PKCα was increased in prostate tissues of CNP rat models, which was reversed by FTA. The above findings implied that the inhibiting effect of FTA on NF-κB activation in CNP rat models could be mediated by targeting PKCα. Notably, this study has some limitations, as it did not provide direct visual evidence of nuclear translocation through immunofluorescence microscopy. Future studies could incorporate immunofluorescence techniques to more clearly elucidate the spatial and temporal resolution of this critical cellular process.

In summary, this study provides new evidence that FTA alleviates inflammation and facilitates autophagy in CNP rat models by blocking the PKCα/NF-κB pathway. On the basis of our current findings, we propose that FTA holds promise as a potential therapeutic agent for CNP.

## Data Availability

The original contributions presented in the study are included in the article/supplementary material, further inquiries can be directed to the corresponding author.
